# Editorial: Understanding Gamma Delta T Cell Multifunctionality - Towards Immunotherapeutic Applications

**DOI:** 10.3389/fimmu.2020.00921

**Published:** 2020-05-14

**Authors:** Kenth Gustafsson, Thomas Herrmann, Francesco Dieli

**Affiliations:** ^1^Department of Biochemical Engineering, University College London, London, United Kingdom; ^2^Institute for Virology and Immunology, Julius Maximilians University Wurzburg, Wurzburg, Germany; ^3^Central Laboratory of Advanced Diagnosis and Biomedical Research, University of Palermo, Palermo, Italy; ^4^Department of Biomedicine, Neurosciences and Advanced Diagnosis, University of Palermo, Palermo, Italy

**Keywords:** γδ T cells, γδ T cell receptor, antigen recognition, killing mechanisms, infectious diseases, tumor immunology

## Introduction

γδ T cells have been characterized by the expression of a γδ T cell receptor (TCR). When the γδ TCR and the corresponding αβ TCR were first discovered it was assumed that the corresponding cell types were likely to be functionally very similar. However, some 30 years later, we have realized that they are not. Unlike αβ T cells, γδ T cells (i) sense target antigens independent of MHC molecules; (ii) display NK-cell like innate reactivities, including killing of infected cells as well as microbes; (iii) are able to take up large particulates, including bacteria, and (iv) can act as professional antigen presenting cells.

The “stress sensing” abilities of γδ T cells have led to a great interest in exploring their potential use in novel immunotherapies, not least in cancer. In addition, their capacity to produce various cytokines and to interact with other cells, such as lymphocytes, myeloid cells, and neutrophils, has raised an interest in their potential therapeutic use as immunomodulators. γδ T cells have therefore recently come to stand out as a distinctly unique cell type. At the same time, we have come to realize that γδ T cells are likely to be an ancient cell type with ancestors as far back as in our common ancestors to jawless fish and thereby, potentially predating the development of our adaptive immune system.

It is clear that to make full use of the great potential of γδ T cells in immunotherapeutic interventions, we must significantly improve our understanding of the extensive plasticity and multifunctionality of γδ T cells and of how these cells can be harnessed therapeutically, both safely and effectively.

This Research Topic, contains 26 articles which includes Review, Original Research, and Methods articles in the area of γδ T cell plasticity and multifunctionality, As shortly overviewed in this editorial, these articles shall not represent entire field of γδ T cell biology but primarily focuses on how an increased knowledge in this area can be used and developed further toward improved immunotherapeutic applications in cancer, infectious disease, autoimmunity, and other immunity-related areas.

## γδ T Cells at Barriers

γδ T cells and other “non-conventional” T cells V-gene usage of antigen-receptors often correlates with distinct developmental and functional features. A prominent example is murine dendritic epidermal γδ T cells (DETC). They carry a single “canonical” γδ TCR and depend on the butyrophilin-like Skint-1 molecule for development. Humans lack DETC as Skint-1 is a pseudogene. The mini review of Sutoh et al. discusses the evolution of the Skint-1 family and DETC like cells. They identify two species with a functional Skint-1 gene, namely cattle and the cynomolgous macaque, as species with skin γδ T cells sharing some features with murine DETC. Interestingly, in lamprey, VLRC-expressing cells (one of the three linages of VLR-antigen-receptor expressing lymphocytes) localize to epithelia and exhibit a dendritic morphology in the skin. This led to the hypothesis that stress-sensing lymphocyte at body surfaces provide an evolutionary advantage and might even have existed in the ancestor of jawed- and jawless-vertebrates.

The interplay of DETC and dermal Vγ4 T cells during wound healing is the focus of two papers by the group of Li, Wang et al. and Li, Wu et al.. They review current knowledge on this subject and provide an original research paper which investigates how epidermal invasion of IL-17 producing dermal Vγ4 cells suppresses IGF-1 production by DETC and wound healing via IL-23 and IL-1β producing keratinocytes.

Cruz et al. provides a comprehensive review on immune responses and pathophysiology of the human skin T cells. Although their cellular composition is quite different from that of mice, it is quite intriguing that many molecular players, especially cytokines, may function similarly as in mouse models. A challenge is now to understand the relative contribution of the different T cell subsets, including γδ T cells, to integrity of healthy skin and their function in pathological conditions such as psoriasis, alopecia areata, type II diabetes and melanoma.

The mini review of McCarthy and Eberl covers the two major subsets of human γδ T cells in mucosal immunity and inflammation: stress-responsive, partially resident Vδ1 lymphocytes and microbial phosphoantigens-sensing Vγ9Vδ2 T cells. Both γδ T cell subsets activate other (resident) leukocytes such as neutrophils and myeloid DCs or differentiate to professional APC, as shown for phosphoantigen-activated Vγ9Vδ2 T cells.

The authors discuss how in both situations γδ T cells help to communicate their findings of stressed transformed and infected cells as well as extracellular pathogens downstream to other immune cells. They speculate how this leads to inflammatory responses and T cell activation serving as the boundaries of our insides and the environment and how this knowledge can be used for development of new therapeutic strategies.

Reinhardt and Prinz, discuss the pathogenesis of various forms of spondyloarthritis. Currently, first choice of Spondylitis ankylosans treatment is non-steroidal anti-inflammatory drugs, physiotherapy and anti-TNF but more recently genome wide association studies and promising results with antibodies targeting IL-17 suggest the involvement of IL-17, IL-22, and IL-23. The authors develop a scenario in which enthesis-resident cells sense directly or indirectly mechanical stress and various innate T cells (e.g., MAIT cells or γδ T cells) and/or ILC3 and promote joint inflammation by production of IL-17 and IL-22.

## γδ T Cells in Autoimmunity and Infection

γδ T cells are abnormally regulated in some autoimmunities. In an original research article Migalovich Sheikhet et al. show that systemic sclerosis (SSc) γδ T cell subsets are differentially regulated by cardiolipin and zoledronate as compared to healthy donor (HC) γδ T cells. CD25^+^ γδ T cells were elevated significantly in SSc patient blood compared to HC. The main conclusions and take home messages of this study was that SSc γδ T cell functional responses were abnormal when stimulated by cardiolipin and phosphoantigens and that this may contribute to fibrosis and immunosuppression, both hallmarks of this disease.

Whilst novel forms of cancer immunotherapies is one of the dominating fields of current research and proposed uses of γδ T cells in the clinic, most investigators in the field would agree that γδ T cells, as indeed other parts of the immune system, evolved to combat pathogens. It is in this spirit we have included three articles in this section describing functional involvement of γδ T cells in infectious diseases.

It is well-known that as HIV virus infect CD4^+^ T-cells, the pool of T cells that are able to produce IL-17 are reduced. γδ T-cells can recognize and kill cells that are infected with virus, including cells infected with HIV virus. As γδ T cells, and in particular Vδ1 γδ T cells, are also able to produce IL-17, they therefore provide an alternative source for IL-17 production in AIDS. However, in an original research paper in this section, Dunne et al. show that such Vδ1 γδ T cells in HIV infected individuals are often exhausted. They show that this exhaustion is likely to be mediated by either PD-1 induction or by downregulation of CD3ε expression.

Malaria is caused by the protozoan parasite *Plasmodium* sp. As malaria causes high mortality and morbidity on several continents and no successful vaccine exists malaria research should be a priority. In a review article in this section Howard et al. summarize what is known about the participation of Vγ9Vδ2 T cells in malaria immune system reactivity. It is well-known that this γδ T cell subset is activated and expanded during a primary *Plasmodium* infection, but not why and what functions this expansion mediates. Following a review of what is known about this subsets innate and adaptive functions, Howard et al. speculate that this subset could serve an important role as an adjuvant in new malaria vaccine formulations.

In an accompanying minireview, Hviid et al. summarize what is known about the participation and possible functions of the Vδ1 γδ T cell subset. They suggest that the more “adaptive” immunological behavior of this subset, as compared to Vγ9Vδ2 T cells, is very likely reflected in their participation in malaria immune reactivity and that this could also be the case for other γ/δ chain combinations other than Vγ9Vδ2.

## γδ T Cells in Tumor Immunity

Pioneering studies by Hayday and colleagues in a mouse model of cutaneous carcinogenesis have firmly established an important role of γδ T cells in immune surveillance (1). Subsequently, it has been found that human γδ T lymphocytes display potent HLA-unrestricted cytotoxicity *in vitro* against a variety of tumor cell lines and have demonstrated efficacy *in vivo* when transferred into immunodeficient mice xenografted with different types of tumor cells.

Given their HLA non-restricted method of antigen recognition, the role of γδ T cells in anti-tumor immunity has stimulated great interest to explore their potential for cancer immunotherapy. Interestingly, the production of endogenous PAgs, such as IPP, can be pharmacologically manipulated by aminobisphosphonates (N-BP), such as Zoledronate, which inhibit FPPS, the downstream enzyme of the MVA pathway, leading to accumulation of endogenous IPP and Vγ9Vδ2 T cell activation.

Simões et al. summarize current knowledge on target cell recognition of γδ human T cells. Antigens expressed by target cells are (over) expressed in stressed or transformed cells. Many of them are unknown and knowledge on the molecular basis of their recognition by the γδ TCR is very limited. Vγ9Vδ2 T-cells sense phosphoantigens, isoprenoid synthesis metabolites, which accumulate as after administration of drugs (amino-bisphosphonates), cell-transformation or microbial infections. This sensing is mediated by the Vγ9Vδ2 TCR which “sees” target cells after binding of the phosphoantigens to target cell-expressed butyrophilin (BTN)3A1 molecule. BTN and butyrophilin-like (BTNL) molecules mainly present on epithelial and tumor cells regulate the homing and maturation of certain γδ clonotypes. BTN3A1 is required for Vγ9Vδ2 T cell recognition of PAgs and BTN3A1 modulation *in vitro* and *in vivo* impacts on anti-tumor efficacy of γδ T cells (Blazquez et al.), thus providing the potential for novel Vγ9Vδ2 T cell-based cancer immunotherapy.

Activating NK cell receptors with NKG2D as most prominent example, act either alone or by providing a co-stimulatory signal to the γδ TCR. They serve also as stress-sensors since expression of many NKG2D ligands results from cell-stress (2). Similar to NK cells, γδ T-cells are endowed with anti-leukemia and anti-infection potential and do not mediate graft-vs.-host disease. These features are particularly useful in the setting of HLA haploidentical HSCT depleted of αβ^+^ T and B lymphocytes to cure high-risk acute leukemias (Pistoia et al.). In this setting, high numbers of both γδ T cells (Vδ1 and Vδ2) are infused together with CD34^+^ HSC and may contribute to rapid control of infections and leukemia relapse. In addition, Zoledronate potentiates the cytotoxic activity of γδ T cells *in vitro* and its infusion in patients strongly promotes γδ T cell differentiation and cytolytic activity. Hence, treatment with Zoledronate may contribute to further improve the patient clinical outcome after HLA-haploidentical HSCT depleted of αβ^+^ T and B lymphocytes.

Intravenous application of Zoledronate together with low-dose IL-2 has been evaluated as a means of *in vivo* activation of γδ T cells in cancer patients. Hoeres et al. provide a comprehensive review of established and newer strategies exploiting γδ T cells in cancer immunotherapy.

Most likely, strategies aiming to activate γδ T cells *in vivo* will have to be combined with other treatment regimens to obtain optimal anti-tumor activity. Bhat et al. discuss the current development of drugs targeting major pathways of epigenetic regulation and their possible impact on γδ T cell multifunctionality and develop concepts of how some of these approaches might help to improve the efficacy of γδ T cell-based cancer immunotherapy.

Additional strategies to improve the anti-tumor activity of γδ T cells are under study.

These include the use of antibodies to trigger Fc receptor-dependent ADCC, or the use of bispecific antibody constructs to cross-link the γδ TCR with tumor cell surface antigens. Varesano et al. have used the therapeutic anti-EGF-R humanized mAb Cetuximab, in addition to Zoledronate in order to enhance Vδ2 T cell-mediated killing of cancer cells. For this purpose, they have employed a 3-D culture system, consisting of colorectal cancer spheroids, and have shown that Cetuximab triggers Zoledronate-activated Vδ2 T cells to perform ADCC of colorectal cancer and destroy spheroids. Thus, this 3-D system may prove reliable to evaluate the whole anti-tumor effect of combinatorial immunotherapies.

γδ T cells can be redirected to the cancer cell using antibodies. This can be achieved, for instance by the use of bispecific antibodies, in which one binding site recognizes a tumor specific cell surface molecule and the other binding site recognizes CD16 or CD3 or the Vγ9Vδ2 TCR; such bispecific antibodies have shown efficacy in preclinical mouse models. Oberg et al. employed the bispecific antibody HER2xCD16 in the form of a “*tribody*” construct to redirect CD16 expressing γδ and NK cells against HER2 positive tumor cell targets. Compared to Trastuzumab, the HER2xCD16 tribody had superior efficacy in promoting CD16-mediated γδ and NK killing of several types of tumor cells expressing HER2 antigen.

Another approach to improve the efficacy of γδ T cell-based cancer immunotherapy consists in the adoptive transfer of genetically-modified T lymphocytes. This includes lentiviral-mediated transduction of T cells with chimeric antigen receptors (CARs) or with an exogenous TCR of known specificity. To date, both approaches have utilized αβ T cells, but γδ T cells may also be an appealing target. Here, Fisher and Anderson provide a comprehensive review of current engineering approaches in human γδ T cells for cancer immunotherapy. Thus, a tumor-specific αβ TCR can be introduced into γδ T cells without the risk of mispairing between the endogenous and exogenous TCRs. It is also possible to transduce peripheral T lymphocytes with a high-affinity Vγ9Vδ2 TCR (Straetemans et al.). These engineered T cells, named TEGs (T cells engineered to express a defined γδT cell receptor), are broadly reactive against several different tumors and have been produced under GMP conditions to enter a Phase I clinical trial in patients with relapsed/refractory acute myeloid leukemia.

One of the basic observations supporting a role of γδ T cells in tumor immune surveillance is their presence amongst tumor-infiltrating lymphocytes (TIL) in most human tumors.

However, the clinical relevance of γδ TILs is unclear because the relative frequencies of tumor-infiltrating γδ T cells correlate with tumor remission, or with tumor progression or even fail to correlate with prognosis. These findings are strongly suggestive of the fact that γδ T cells in the tumor microenvironment may play opposite functions and thus positive or negative correlation with prognosis may depend on the specific γδ T cell subset present at the tumor site (Pauza et al.). It is clear, however, that an increased presence of γδ T cells within a tumor *per se* is not necessarily associated with a beneficial effect. As discussed here by Lo Presti et al., there are multiple interactions of tumor-infiltrating γδ T cells within the local tumor microenvironment that strongly influence the functional outcome. Relevant factors include (but are not restricted to) tumor-derived immunosuppressive cytokines and metabolites, locally expressed inhibitory checkpoints such as PD-1 (Castella et al.), myeloid-derived suppressor cells (MDSCs, Sacchi et al.) and hypoxia (Siegers et al.). Hence, different conditions occurring in the context of the tumor microenvironment may inhibit γδ T cell functions or even convert γδ T cells into suppressive cells, which negatively influence tumor outcome and patient's prognosis.

Therefore, it is a major challenge for future studies to determine how to specifically boost the anti-tumor effects of γδ T cells while simultaneously shunting their suppressive activity.

## Concluding Remarks

After 30 years of γδ T cell research, it is clear that these cells are intimately involved in the control of tissue homeostasis, infection, and malignancy (Edelblum et al.). The identification of specific ligands for the γδ TCR provides strong support for the idea that γδ T cells are non-redundant to αβ T cells. Apart from the detailed knowledge of their physiological and pathophysiological significance, we are currently experiencing new exciting developments aimed at bringing γδ T cells into clinical medicine.

## Author Contributions

All authors listed have made a substantial, direct and intellectual contribution to the work, and approved it for publication.

## Conflict of Interest

The authors declare that the research was conducted in the absence of any commercial or financial relationships that could be construed as a potential conflict of interest.
